# Improving succinylation prediction accuracy by incorporating the secondary structure via helix, strand and coil, and evolutionary information from profile bigrams

**DOI:** 10.1371/journal.pone.0191900

**Published:** 2018-02-12

**Authors:** Abdollah Dehzangi, Yosvany López, Sunil Pranit Lal, Ghazaleh Taherzadeh, Abdul Sattar, Tatsuhiko Tsunoda, Alok Sharma

**Affiliations:** 1 Department of Computer Science, Morgan State University, Baltimore, Maryland, United States of America; 2 Department of Medical Science Mathematics, Medical Research Institute, Tokyo Medical and Dental University, Tokyo, Japan; 3 Laboratory for Medical Science Mathematics, RIKEN Center for Integrative Medical Sciences, Yokohama, Kanagawa, Japan; 4 School of Engineering & Advanced Technology, Massey University, Palmerston North, New Zealand; 5 School of Information and Communication Technology, Griffith University, Queensland, Australia; 6 Institute for Integrated and Intelligent Systems, Griffith University, Queensland, Australia; 7 CREST, JST, Tokyo, Japan; 8 School of Engineering & Physics, University of the South Pacific, Suva, Fiji; UMR-S1134, INSERM, Université Paris Diderot, INTS, FRANCE

## Abstract

Post-translational modification refers to the biological mechanism involved in the enzymatic modification of proteins after being translated in the ribosome. This mechanism comprises a wide range of structural modifications, which bring dramatic variations to the biological function of proteins. One of the recently discovered modifications is succinylation. Although succinylation can be detected through mass spectrometry, its current experimental detection turns out to be a timely process unable to meet the exponential growth of sequenced proteins. Therefore, the implementation of fast and accurate computational methods has emerged as a feasible solution. This paper proposes a novel classification approach, which effectively incorporates the secondary structure and evolutionary information of proteins through profile bigrams for succinylation prediction. The proposed predictor, abbreviated as SSEvol-Suc, made use of the above features for training an AdaBoost classifier and consequently predicting succinylated lysine residues. When SSEvol-Suc was compared with four benchmark predictors, it outperformed them in metrics such as sensitivity (0.909), accuracy (0.875) and Matthews correlation coefficient (0.75).

## Introduction

Post-translational modification (PTM) refers to the enzymatic modification of proteins [1]. As part of this biological mechanism, one or more amino acids of a protein interact with specific molecular groups. Such interaction functionally changes the amino acids, thereby impacting the function of the entire protein. From the 20 amino acids that make up the genetic code, lysine has proven to be the most susceptible residue to PTM. It has been involved in many PTMs including methylation [2, 3], sumoylation [4], acetylation [5], glycation [6] and malonylation [7]. One of the recently identified PTMs is succinylation, which reportedly impacts the function and structure of proteins within biological interactions [8]. Succinylation mainly refers to the addition of a succinyl group to lysine residues. This molecular change alters the charge of the lysine to -1, thus introducing a large structural moiety. Succinylation occurs in both eukaryotic and prokaryotic cells, and is common in enzymes involved in mitochondrial metabolism, amino acid degradation, and fatty acid metabolism. It has been also observed in histones with functions in chromosome configuration and gene expression. Nevertheless, the role of succinylation in other biological reactions needs to be extensively clarified. Therefore, the identification of succinylation sites can provide detailed insights into the function of proteins and their biological interactions.

The identification of PTM sites has become a serious challenge in the last years [9]. In this direction, many bioinformatics methods have been proposed for detecting them within protein sequences [10–30]. Experimental methods like mass spectrometry remain the main technique for identifying lysine succinylation sites. However, these methods are costly and unable to keep up with the exponential growth of sequenced proteins. Consequently, there is an urgent demand for fast and accurate computational methods capable of predicting succinylation sites. In the past years, a wide range of prediction methods have been proposed to tackle this issue, but their performance is consistently limited. This limitation is more apparent for lysine succinylation than for other PTMs because of its recent discovery. Thus far, the pioneering methods proposed to solve this problem have been mainly focused on analyzing the protein sequence. Two of these methods are SucPred [31] and SuccFind [32]. The former is a semi-supervised machine learning-based method, which incorporates the sequence and physicochemical features into a support vector machine for classification. The later, however, introduced a more robust approach that considers information about the neighboring amino acids of succinylated and non-succinylated lysines to better discriminate between them. Another method, iSuc-PseAAC, employed a strategy that integrates the peptide position-specific propensity into the general form of pseudo amino acid composition for training a support vector machine [33]. Another method that incorporates sequence-coupling effects into the pseudo amino acid composition was iSuc-PseOpt [34]. It introduced the k-nearest neighbors strategy and hypothetical training samples in an attempt to ameliorate the imbalance between classes. Subsequently, a random forest algorithm was designed for prediction. SuccinSite also regarded a random forest classifier but with informative encoding features, such as the composition of k-spaced amino acid pairs, binary encoding and specific physicochemical attributes [35]. However, the above predictors showed a poor sensitivity when it comes to detecting succinylated lysine residues.

Studies related to protein subcellular localization [36], structure and function prediction [37, 38], and local structure and torsion angles prediction [39] have demonstrated that the structural and evolutionary information of proteins can significantly improve prediction performance. We previously proposed two different predictors: SucStruct [40] and PSSM-Suc [41], which corroborated the above premise. For instance, SucStruct used structural features like secondary structure and torsion angles [40], whereas PSSM-Suc transformed the evolutionary information of the position specific scoring matrix (PSSM) for succinylation prediction [41]. Both approaches trained a pruned decision tree for classification purposes, and outperformed state-of-the-art predictors which only relied on sequence and physicochemical attributes. These predictors clearly demonstrated that the use of powerful classifiers alongside evolutionary and structural attributes can significantly improve succinylation prediction.

In order to design an efficient sequence-based computational predictor for solving biological problems, a long list of studies [25, 26, 42–47] has made reference to a five-step rule [48]. This rule comprises the following steps: (1) the construction or selection of a correct dataset for training and testing a predictor, (2) the use of an accurate mathematical expression for transforming the biological sequence and considering the intrinsic correlation to future predictions, (3) the development of an exact algorithm for making predictions, (4) the proper use of statistical metrics for assessing the predictor accuracy, and (5) the design of a user-friendly server for making the predictor available to the public. These steps will be described in the subsequent sections.

Given the explosion of biological sequences, one of the most serious challenges is how to represent these sequences as discrete models or vectors while keeping the information related to the order of sequences. This problem is often caused by the intrinsic limitations of machine learning algorithms, which can only handle numerical vectors [16]. Besides any vector could lose the information of patterns in a sequence. In order to overcome the above limitations for protein sequences, the pseudo amino acid composition (PseAAC) [49] was proposed. Since its proposal, the concept known as Chou’s PseAAC, has been widely used in the area of computational proteomics [9, 50]. PseAAC has been recently incorporated in three software: ‘PseAAC-Builder’, ‘propy’, and ‘PseAAC-General’. The first two are aimed at creating models of Chou’s special PseAAC, whereas the third one uses the Chou’s general PseAAC [48]. These software considered the special modes of feature vectors in addition to high-level vectors such as ‘functional domain’, ‘gene ontology’ and ‘sequential evolution’, or ‘PSSM’ modes [48]. Due to the usefulness of PseAAC for dealing with protein/peptide sequences, a new concept coined pseudo k-tuple nucleotide composition [51], aimed at generating feature vectors from DNA/RNA sequences, was proposed. Recently, a new web server called ‘Pse-in-One’ [52] and its updated version ‘Pse-in-One 2.0’ [53], which facilitate the generation of feature vectors from protein/peptide or DNA/RNA sequences, were developed. Our study made use of secondary structure and evolutionary information for defining pseudo components and thus identifying succinylation sites.

In this work, we propose a new predictor, SSEvol-Suc, which primarily integrates information about the best secondary structure and the PSSM for predicting succinylation sites [54, 55]. Our predictor combines both features and transforms them into profile bigrams [56] in order to describe each lysine residue. The k-nearest neighbors strategy was employed for reducing the imbalance between succinylation and non-succinylation sites [34]. An AdaBoost classifier was finally designed for discriminating between lysine residues. We compared the prediction results of SSEvol-Suc with those of iSuc-PseAAC [33], SuccinSite [35], iSuc-PseOpt [34] and pSuc-Lys [57]. SSEvol-Suc achieved remarkable results by outperforming all the above predictors. Its sensitivity, accuracy and Matthews correlation coefficient (MCC) were recorded at 0.909, 0.875 and 0.75, respectively.

## Materials and methods

In this paper, we propose a novel predictor, SSEvol-Suc, which makes use of the secondary structure and the PSSM of proteins for accurately predicting succinylation sites [58–60]. These features were transformed into profile bigrams and employed for describing each lysine residue. The resulting matrix was then used for training an AdaBoost classifier and predicting succinylated lysines.

### Benchmark dataset

The benchmark dataset was extracted from the Compendium of Protein Lysine Modifications (CPLM) [61, 62]. This compendium consists of over 45,000 proteins from 122 species, and 12 different annotated PTMs. In the CPLM, succinylation was the most abundant and diversely distributed mark across all the included species [62]. The original collection comprised 2,521 succinylation and 24,128 non-succinylation sites from 896 unique proteins. To avoid overestimations due to homology and be able to directly compare our results with those of previous studies, we removed those proteins with ≥ 40% pairwise sequential similarity. We then ended up with a benchmark dataset consisting of 670 unique proteins, where the longest and shortest proteins were 5,656 and 47 residues long and the average protein comprised 464 residues. The 1,782 succinylation and 18,344 non-succinylation sites located in such proteins were grouped into two mutually exclusive collections: positive and negative. The subsequent sections will introduce the structural and evolutionary features computed from the protein sequences.

### Secondary structure feature

The secondary structure of proteins provides accurate information about their local structure and how they fold into their general tertiary configuration. We predicted the secondary structure of each protein in our benchmark dataset with the tool SPIDER2 [63, 64]. SPIDER2 is one of the latest predictors aimed at computing the local structure of proteins. This software has been successfully used to compute the structural properties of proteins in sequence-based predictions of protein binding sites. Secondary structure indicates the contribution of each amino acid to specific local structures, namely, helix, strand, and coil for determining the local 3D structure of proteins. In other words, secondary structure determines the local structure of proteins by considering the local configuration of amino acids in the sequence. Therefore, its understanding can provide critical information about the function and folding of proteins. We run SPIDER2 on each protein sequence by providing all the sequences in FASTA format. SPIDER2 automatically detects a FASTA file, and for each sequence, it retrieves the local structure with the highest probability. This results in a matrix of size *L* × 3, where *L* represents the protein length and the three columns indicate the transition probabilities to the three secondary structure conformations (helix, strand and coil). Hereafter, we refer to this matrix as *SSpre*.

### Evolutionary feature

Evolutionary information provides valuable insights into structural, functional and sequential similarities among proteins based on how they evolved [65]. PSSM describes the substitution probability of each amino acid in a protein with all the amino acids of the genetic code. This matrix was computed with the alignment toolbox PSI-BLAST [66], which aligns each protein to similar proteins in the Protein Data Bank [67]. We run PSI-BLAST on all the proteins in our benchmark dataset and retrieved the corresponding PSSM. For each protein, PSI-BLAST produces two *L* × 20 matrices, where *L* is the protein length and the 20 columns indicate the amino acids of the genetic code. The running of PSI-BLAST was conducted on non-redundant proteins in the Protein Data Bank, with a cutoff (E) of 0.001 and three iterations. From these matrices, we used the normalized matrix which comprises the substitution probabilities of amino acids.

### Lysine residues as profile bigrams

**Fig 1:**
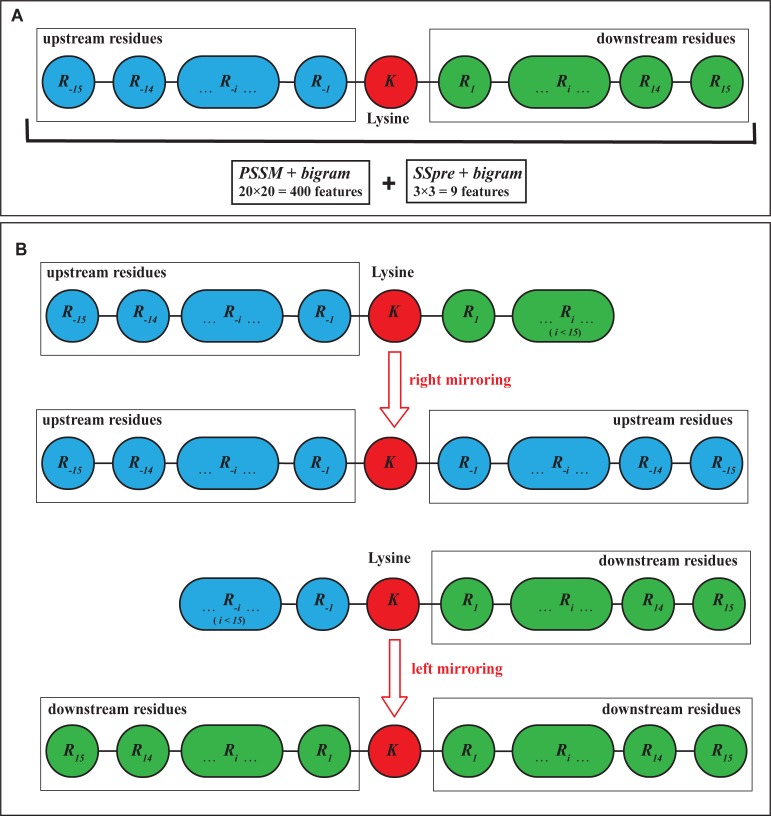
Schematic representation of a lysine residue and its surrounding amino acids. (A) lysine with 15 residues on both sides, (B) lysine with missing residues to the right and left.

The structural and evolutionary features were used to describe each succinylated and non-succinylated lysine residue. Lysines (*K*) were described by considering their adjacent 15 upstream and 15 downstream amino acids (Fig 1A) [34]. If a lysine residue did not contain 15 amino acids (either upstream or downstream), we mirrored the missing peptide stretch (Fig 1B). The sequence segment *S* consisting of 15 upstream and 15 downstream residues in addition to the lysine *K* was expressed as

$$S=\{R_{-15},R_{-14},\ldots ,R_{-2},R_{-1},K,R_{1},R_{2},\ldots ,R_{14},R_{15}\}$$ (1)

where *R*_−*i*_ and *R*_*i*_ (for 1 ≤ *i* ≤ 15) are upstream and downstream amino acids, respectively. It can be observed from Eq (1) that 31 amino acids (including *K*) were used for defining each lysine residue. Accordingly, each lysine represented by the sequence segment *S* was labeled. In other words, the segment *S* comprising a succinylation site was labeled as 1 whereas that describing a non-succinylation site was labeled as 0.

To describe each lysine, the submatrices *SSpre* and *PSSM* around the lysine were retrieved and transformed into frequency vectors of bigrams (*PSSM* + *bigram* and *SSpre* + *bigram*). These transformations resulted in two matrices of sizes 20 × 20 (for *PSSM* + *bigram*) and 3 × 3 (for *SSpre* + *bigram*). Each segment *S* was finally described by a 409-feature vector. This feature vector was then used to capture the structural and evolutionary information about the lysine represented by the segment *S*.

The bigram method has shown promising results when it comes to exploring discriminatory information [36, 56, 68–70], so that we used it here. The scheme for transforming the submatrices *SSpre* and *PSSM* into frequency vectors is explained below. The *PSSM* matrix *M* of size *L* × 20 and the *SSpre* matrix *N* of size *L* × 3 were used to construct a feature vector. Each element *m*_*ij*_ and *n*_*ij*_ of the matrices *M* and *N*, respectively, represents the transitional probability of the *j* -th amino acid/secondary structure conformation at *i*-th position in the protein sequence. The sequence segment *S* (Eq (1)) was described by two matrices of sizes 31 × 20 (for *PSSM*) and 31 × 3 (for *SSpre*). The matrices *M* and *N* were processed as profile bigrams [56] by

$$B_{p,q}=\sum_{k=1}^{30}m_{k,p}m_{k+1,q}$$ (2)

and

$${B\prime }_{r,s}=\sum_{k=1}^{30}n_{k,r}n_{k+1,s}$$ (3)

where 1 ≤ *p*,*q* ≤ 20 for the matrix *M* and 1 ≤ *r*,*s* ≤ 3 for the matrix *N*.

Thus, the matrix *B*, which represents *PSSM* + *bigram* and its elements *B*_*p*,*q*_ (for *p* = 1,2,…,20 and *q* = 1,2,…,20), will be a 20 × 20 matrix. Similarly, the matrix *B*′, which represents *SSpre* + *bigram* and consists of elements $B_{r,s}^{\prime }$, (for *r* = 1,…,3 and *s* = 1,…,3) will be of size 3 × 3. Subsequently, the matrices *B* and *B*′ can be transformed as

$$F={\left[B_{1,1},B_{1,2}\ldots ,B_{1,20},B_{2,1},\ldots B_{20,1},\ldots B_{20,20},{B^{\prime }}_{1,1},B_{1,2}^{\prime }\ldots ,{B\prime }_{3,3}\right]}^{T}$$ (4)

where *T* is the transpose. Therefore, the matrix *B* will have 400 transitional probabilities and the matrix *B*′ will comprise 9 transitional probabilities. Eq (4) is the feature vector, which contains 409 transitional probabilities and results from the *PSSM* + *bigram* and *SSpre* + *bigram* matrices. In other words, each lysine residue was defined by a 409-dimensional vector of structural and evolutionary features.

This information was computed for all the lysine residues in our benchmark dataset, resulting in a training matrix of 1,782 succinylation sites (*label* = 1) and 18,344 non-succinylation sites (*label* = 0). Such a matrix was further processed to reduce the imbalance between classes, and ultimately used for training an AdaBoost classifier (refer to the following section).

One advantage of the bigram method is its window-size independent nature. For instance, it extracts 400- and 9-dimensional feature vectors regardless of the window size adopted around lysine residues. Thereby, the bigram method enables us to enlarge the window around lysines without necessarily increasing the number of features.

### AdaBoost classifier

Adaptive Boosting (AdaBoost) is a meta-classifier, which iteratively applies a base learner and adjusts its parameters to build a strong ensemble classifier [71]. The base classifier, usually a decision tree, is first applied to the training dataset. The weights are then iteratively adjusted by increasing the weight for misclassified samples. This procedure continues until changes in the weights become trivial. Finally, AdaBoost combines the base classifiers across all the iterations to build the final predictor [72]. Decision trees are usually used as base classifiers because they can reflect larger changes due to their sensitivity to weight adjustments [72, 73]. AdaBoost has been successfully used in studies related to protein folding, attaining promising results that emphasize its applicability to protein science [74, 75]. We utilized the Weka implementation of the AdaBoost algorithm [76] with 1,000 iterations. Decision stumps, which are one-level decision trees, were used as weak classifiers.

## Results and discussion

Any predictor, aimed at predicting succinylation sites, must have its performance assessed. In this work, we evaluated the performance of SSEvol-Suc in terms of four different statistical metrics: sensitivity, specificity, accuracy and Matthews correlation coefficient [15, 36, 77–80]. The following sections will discuss these metrics in addition to aspects such as class imbalance and predictor performance.

### Evaluation metrics

The first metric, sensitivity, was used to evaluate the proportion of correctly predicted succinylation sites. If the predictor is able to accurately detect succinylation sites in the dataset, a high sensitivity will be achieved. For instance, a predictor with a sensitivity of 1 is able to accurately detect positive (succinylation) sites whereas that with a sensitivity of 0 fails to detect these sites.

The second metric, specificity, assesses the predictor ability to correctly detect non-succinylation sites. Similarly, a specificity of 1 presents a predictor able to classify all the negative sites whereas a specificity of 0 points to a predictor unable to detect them.

The third metric, accuracy, evaluates the predictor ability to discriminate between succinylation and non-succinylation sites. The predictor with an accuracy of 1 is an accurate one while that with an accuracy of 0 is regarded an inaccurate predictor.

The fourth metric, Matthews correlation coefficient (MCC), is often used in binary classification when the classes have different sizes. A perfect correlation between observed and predicted instances is indicated by a MCC of 1 whereas a perfect anticorrelation is confirmed by a MCC of -1.

These four metrics can be summarized as

$$sensitivity=1-\frac{N_{-}^{+}}{N^{+}}$$ (5)

$$specificity=1-\frac{N_{+}^{-}}{N_{-}}$$ (6)

$$accuracy=1-\frac{N_{-}^{+}+N_{+}^{-}}{N^{+}+N^{-}}$$ (7)

$$MCC=\frac{1-(\frac{N_{-}^{+}}{N^{+}}+\frac{N_{+}^{-}}{N_{-}})}{\sqrt{\left(1+\frac{N_{+}^{-}{-N}_{-}^{+}}{N^{+}})(1+\frac{{N_{-}^{+}-N}_{+}^{-}}{N^{-}}\right)}}$$ (8)

where *N*^+^ and $N_{-}^{+}$ represent the total amount of positive (succinylation) sites and the number of positive sites misclassified by the predictor. Likewise, *N*^−^ and $N_{+}^{-}$ indicate the total amount of negative (non-succinylation) sites and the number of negative sites misclassified by the predictor.

A promising predictor should ideally outperform in the above statistical metrics. In any case, it should achieve a high performance in at least one of the statistics. Conversely, a predictor with a low sensitivity will be clearly displaying an inability to accurately predict succinylation sites.

### Validation scheme

For assessing the performance of any predictor, the use of an appropriate validation scheme is absolutely necessary. Several validation schemes, including the *n*-fold cross-validation and the jackknife, have been proposed [81, 82]. While the jackknife resampling model turns out to be the least arbitrary and yield unique results for a dataset [83], the cross-validation strategy has been extensively used to evaluate previous predictors [33, 34]. Therefore, we also used the cross-validation scheme here for establishing a fair comparison with state-of-the-art predictors.

The cross-validation technique was carried out as follows,

1. The initial dataset was split into *n* different subsets of equal size.
2. The predictor was trained on the *n* − 1 subsets and tested on the remaining fold.
3. The predictor parameters were adjusted with the *n* − 1 subsets.
4. The four statistical metrics (sensitivity, specificity, accuracy and MCC) were calculated on the test fold.
5. Steps 1 to 4 were repeated *n* times and the average of each statistical metric was computed.

In this study, we assessed the performance of SSEvol-Suc with 6-, 8- and 10-fold cross-validations.

### Dataset balancing

After retrieving the succinylated and non-succinylated lysines from each protein sequence, we obtained a number of non-succinylation (negative) sites greater than that of succinylation (positive) sites. Although such a difference makes sense from a biological viewpoint, it could strongly bias any computational predictor. Because of this, the elimination of class imbalances in training datasets proves critical in pattern recognition studies for achieving bias-free classifications. It is worth noting that different techniques have been proposed for balancing datasets. Though the upsampling of the positive set might further improve the predictor performance as previously suggested [84], we chose to downsample the negative set in order to avoid introducing artificial training instances. Therefore, we used the k-nearest neighbors classifier [34]. To do this, we initially calculated the Euclidean distance between all the instances (lysine residues) in our benchmark dataset. Subsequently, we set a threshold of 10, which indicates the number of neighbors to be regarded. This cutoff, used for ameliorating the imbalance between classes, was intended to provide a better comparison with benchmark predictors [34], which have utilized the same value for dataset balancing. It was computed as the division between the amount of negative (18,344) and positive (1,782) lysines. As a result, those non-succinylation sites, whose 10 nearest neighbors included at least one succinylation site, were removed. However, this initial filtering did not completely eliminate the imbalance so that new cutoffs were computed. These thresholds were calculated by multiplying the initial threshold (*k* = 10) by different integers. The computation procedure was repeatedly carried out until both sets (succinylation and non-succinylation sites) were balanced. Consequently, the number of negative instances was reduced to 1,604 sites with a cutoff of 60 (i.e., non-succinylation sites, whose 60 nearest neighbors comprised at least one succinylation site, were eliminated). The remaining sets were then used to perform cross-validation and evaluate the performance of the proposed predictor.

### Comparison of SSEvol-Suc and current predictors

The proposed predictor, SSEvol-Suc, was compared with four state-of-the-art predictors: iSuc-PseAAC [33], iSuc-PseOpt [34], SuccinSite [35] and pSuc-Lys [57]. These four predictors were implemented into user-friendly web servers for succinylation site prediction. Thereby, we manually uploaded all the protein sequences to the web servers and retrieved their predictions for performance assessment. These web servers were previously trained on part of our sequence dataset that is why we could only compute their performances on the validation set. While the area under the curve (AUC) of iSuc-PseAAC [33], iSuc-PseOpt [34], SuccinSite [35] and pSuc-Lys [57] could not be computed, that of SSEvol-Suc was calculated for 6-, 8- and 10-fold cross-validations.

**Fig 2:**
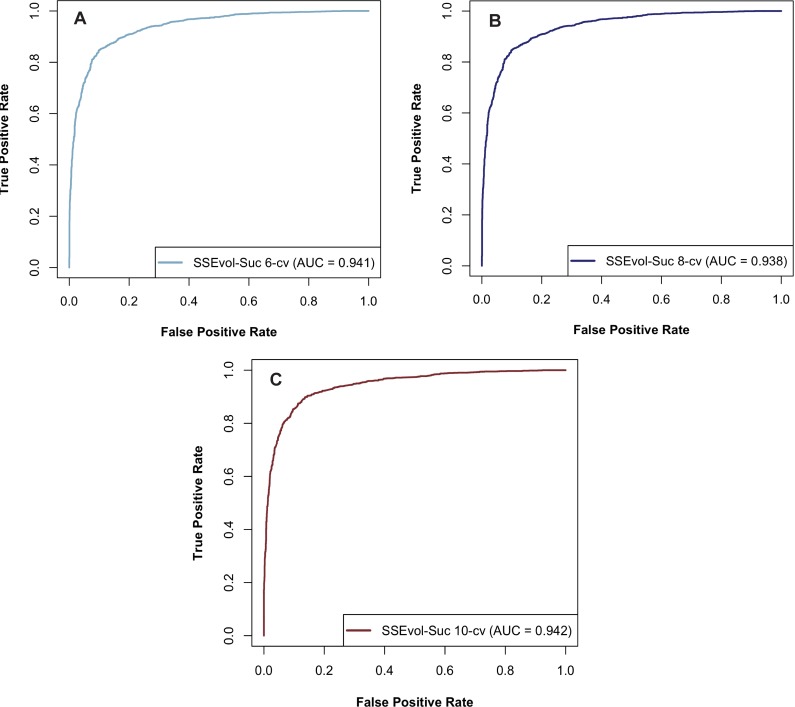
Receiver operating characteristic of SSEvol-Suc for (A) 6-, (B) 8- and (C) 10-fold cross-validations.

As shown in Table 1, SSEvol-Suc represents a significant improvement over the four predictors: iSuc-PseAAC [33], iSuc-PseOpt [34], SuccinSite [35] and pSuc-Lys [57]. SSEvol-Suc outperformed the previous predictors in statistics such as sensitivity, accuracy and MCC. For instance, sensitivity, accuracy and MCC significantly improved by 47.8%, 21.7% and 60.3%, respectively, when compared to the highest value of each metric. These results clearly indicate a considerable improvement (i.e., an increase in succinylation prediction accuracy) over current predictors. It is worth noting that although the specificity (0.906) of SuccinSite [35] remained high, its sensitivity (0.302) was remarkably low, leaving approximately 70% of succinylation residues undetected. In addition, the AUC of SSEvol-Suc for 6-, 8- and 10-fold cross-validations was 0.941, 0.938 and 0.942, respectively (Fig 2). These AUC values show that the predictor performance was not significantly affected when 6- and 10-fold cross-validations were conducted. However, the AUC value tended to slightly decrease when 8-fold cross-validation was performed.

**Table 1:** Comparison of SSEvol-Suc and state-of-the-art predictors.

Method	Sensitivity	Specificity	Accuracy	MCC	AUC
iSuc-PseAAC [33]	0.163	0.873	0.500	0.052	-
iSuc-PseOpt [34]	0.615	0.782	0.694	0.401	-
SuccinSite [35]	0.302	0.906*	0.588	0.258	-
pSuc-Lys [57]	0.587	0.866	0.719	0.468	-
SSEvol-Suc (6-CV)	0.900	0.835	0.870	0.739	0.941
SSEvol-Suc (8-CV)	0.905	0.836	0.872	0.745	0.938
SSEvol-Suc (10-CV)	0.909*	0.837	0.875*	0.750*	0.942*

*Highest value of this metric.

Furthermore, we randomly created 100 negative sets of 1,782 samples each and trained the AdaBoost classifier to properly sample the non-succinylation space. Nevertheless, the average statistical metrics for 6-, 8- and 10-fold cross-validations did not dramatically vary (S1 File).

The above results illustrate the applicability of SSEvol-Suc when it comes to discriminating between succinylation and non-succinylation sites. These could be achieved by the effective combination of secondary structure and evolutionary information about proteins. The information on each peptide segment around a lysine was transformed into matrices of profile bigrams, and finally combined into a feature vector of transitional probabilities for classification purposes. This transformation appears to be essential to detect succinylated lysines and improve the sensitivity of SSEvol-Suc. Besides the AdaBoost classifier also contributed to such prediction outcomes. In summary, the use of one single vector, which combines *PSSM* + *bigram* and *SSpre* + *bigram*, seems to retain necessary information about lysine residues and therefore enables us to accurately detect succinylation sites.

Structural and evolutionary information has been previously considered in two computational predictors [40, 41]. For instance, SucStruct included the *SSpre* feature [40] whereas PSSM-Suc only regarded information about the *PSSM* [41]. Although evolutionary information allowed us to discriminate lysines, better results are achieved when both types of characteristics are combined rather than independently used.

As stated in [85] and demonstrated in numerous studies [10–14, 17, 20–22, 25, 26, 29, 30, 42–44, 47, 86, 87], the availability of user-friendly web servers should be the next step in every computational predictor in order to enhance its impact [9]. To accomplish this, we will intend to build such a web server in the future so that the scientific community could significantly benefit from the proposed predictor.

Additional material related to this study can be downloaded from https://github.com/YosvanyLopez/SSEvol-Suc.

## Conclusions

In this paper, we present a novel predictor called SSEvol-Suc which effectively uses a combination of *PSSM* + *bigram* and *SSpre* + *bigram* for predicting succinylated lysine residues. The secondary structure and evolutionary information about proteins was processed using profile bigrams and further integrated into a single vector for classification purposes. The k-nearest neighbors technique was utilized for removing redundant instances, which were finally input into an AdaBoost classifier for succinylation site prediction. When compared with previous approaches, the sensitivity, accuracy and MCC of the proposed predictor significantly improved by 47.8%, 21.7% and 60.3%, respectively. In spite of the significant performance of SSEvol-Suc, it is worth emphasizing that machine learning techniques do not help us understand why some lysines are succinylated and others are not. This is the main disadvantage of such techniques, which do not provide much scientific knowledge.

## Supporting information

S1 File Performance of the AdaBoost classifier on randomly created negative sets using 6-, 8- and 10-fold cross-validations. (XLSX)
